# Protective impact of circulating ω-3 PUFAs on sepsis susceptibility through *PCSK9* variant: evidence from Mendelian randomization and animal experiments

**DOI:** 10.3389/fmed.2025.1701207

**Published:** 2026-01-13

**Authors:** Qi Han, Zhengxuan Li, Qilong Song, Simeng He

**Affiliations:** 1 Department of Emergency Medicine, Qilu Hospital of Shandong University, Jinan, China; 2 Shandong Provincial Clinical Research Center for Emergency and Critical Care Medicine, Chest Pain Center, Institute of Emergency and Critical Care Medicine of Shandong University, Qilu Hospital of Shandong University, Jinan, China; 3 Medical and Pharmaceutical Basic Research Innovation Center of Emergency and Critical Care Medicine, China’s Ministry of Education, Shandong Provincial Engineering Laboratory for Emergency and Critical Care Medicine, Key Laboratory of Emergency and Critical Care Medicine of Shandong Province, Key Laboratory of Cardiopulmonary-Cerebral Resuscitation Research of Shandong Province, Qilu Hospital of Shandong University, Jinan, China; 4 NMPA Key Laboratory for Clinical Research and Evaluation of Innovative Drug, Qilu Hospital of Shandong University, Jinan, China; 5 The Key Laboratory of Cardiovascular Remodeling and Function Research, Chinese Ministry of Education, Chinese Ministry of Health and Chinese Academy of Medical Sciences, The State and Shandong Province Joint Key Laboratory of Translational Cardiovascular Medicine, Qilu Hospital of Shandong University, Jinan, China

**Keywords:** Mendelian randomization, PCSK9, polyunsaturated fatty acids, sepsis, ω-3

## Abstract

**Background:**

Polyunsaturated fatty acids (PUFAs), particularly ω-3 PUFAs, can improve sepsis prognosis. However, the relationship between the pre-infection levels of PUFAs and sepsis risk remains unclear.

**Methods:**

We conducted a two-sample Mendelian randomization (MR) analysis using UK Biobank data to explore the causal association between circulating unsaturated fatty acids (UFAs) and sepsis susceptibility, complemented by a sepsis mouse model (cecal ligation and puncture, CLP) for validation.

**Results:**

MR analysis revealed that ω-3 PUFAs (OR: 0.912, *p* = 0.005), ω-6 PUFAs (OR: 0.914, *p* = 0.036), total PUFAs (OR: 0.894, *p* = 0.003), and the PUFAs/MUFAs (monounsaturated fatty acids) ratio (OR: 0.927, *p* = 0.042) correlated with a reduced risk of sepsis, while the ω-6/ω-3 ratio increased susceptibility and mortality rates (OR: 1.084, *p* = 0.018). Sensitivity analyses showed no heterogeneity or horizontal pleiotropy, indicating robust findings. Ten single nucleotide polymorphisms (SNPs) were linked to both ω-3 and ω-6 PUFAs. Among which rs11591147 in the *PCSK9* gene (a loss-of-function variant) may mediate the protective effect of PUFAs against sepsis. Mouse experiments showed that ω-3 PUFAs or/and the PCSK9 monoclonal antibody (evolocumab), reduced 7-day mortality (26.7, 40.0, and 53.3%, respectively) and multi-organ damage in CLP mice.

**Conclusion:**

In patients with *PCSK9* mutations, elevated plasma ω-3 PUFAs may reduce susceptibility to sepsis. Therefore, early detection of PUFA levels and PCSK9 genotypes, along with the targeted nutritional supplements, may help reduce sepsis risk in susceptible populations.

## Graphical abstract

**Figure:**
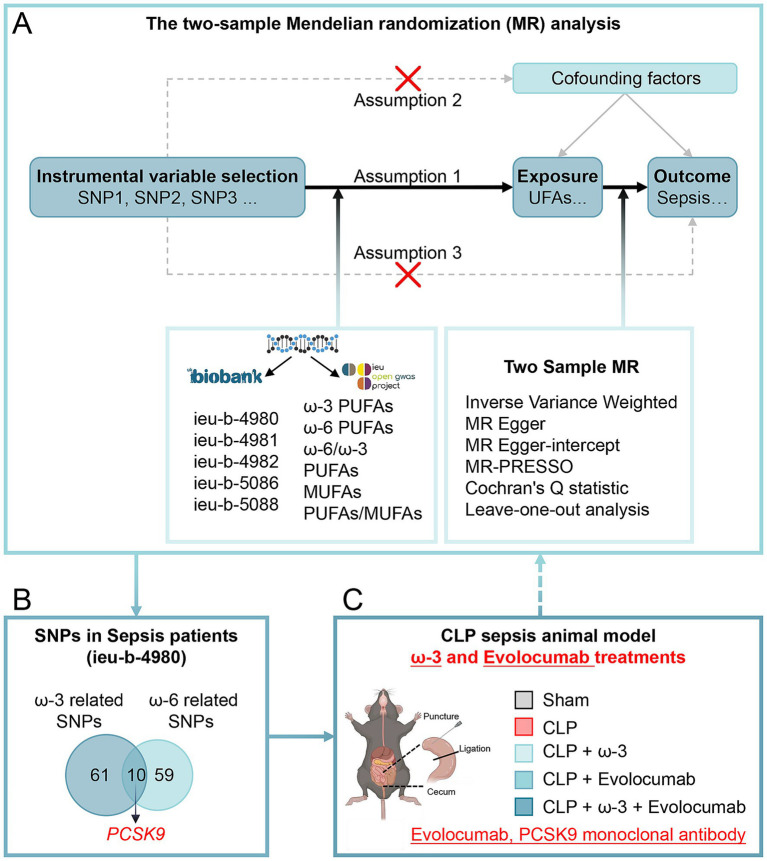
Overview of the study design. **(A)** Two-sample Mendelian randomization analysis was conducted to investigate the causal association between circulating UFAs (including ω-3 PUFAs, ω-6 PUFAs, total PUFAs, total MUFAs, ω-6/ω-3 ratio, and PUFAs/MUFAs ratio) and susceptibility to sepsis. **(B)** Among the 10 SNPs that mediate the occurrence of sepsis and are associated with both ω-3 and ω-6 PUFAs, rs11591147 in the *PCSK9* gene is included. **(C)** CLP mouse experiments demonstrated that both ω-3 PUFAs and the PCSK9 monoclonal antibody, when administered individually or in combination, can significantly reduce mortality and multi-organ damage in sepsis. Partial illustrations were generated using BioRender.com. A valid publication license has been acquired.

## 1 Introduction

**Figure 1:**
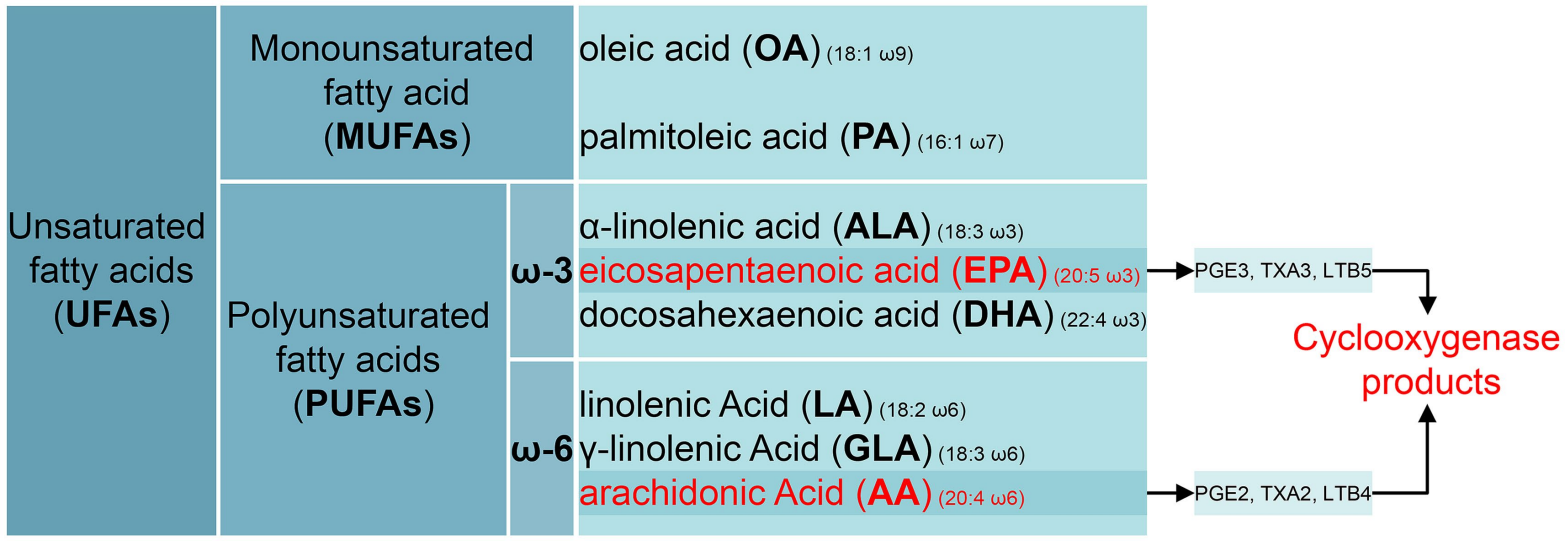
Schematic representation of lipid mediators classes. Unsaturated fatty acids (UFAs) are categorized as either monounsaturated fatty acids (MUFAs) or polyunsaturated fatty acids (PUFAs), based on the number of carbon–carbon double bonds. PUFAs contain two or more double bonds. PUFAs are further classified by double-bond position into series (e.g., ω-3, ω-6, ω-9). Oleic acid (OA) is a ω-9 MUFA, while palmitoleic acid is a ω-7 MUFA. ω-3 PUFAs include alpha-linolenic acid (ALA), eicosapentaenoic acid (EPA), and docosahexaenoic acid (DHA). ω-6 PUFAs include linoleic acid (LA), γ-linolenic acid (GLA), and arachidonic acids (AA). PGE3, TXA3, and LTB5 are derived from EPA, whereas PGE2, TXA2, and LTB4 are derived from AA.

Sepsis is a life-threatening organ dysfunction resulting from host reaction disorders triggered by infection factors (1, 68, 69). Latest data indicate that the global burden of sepsis has increased by 30% due to the COVID-19 pandemic, with an estimated 166 million cases and 21.4 million deaths occurring worldwide annually, accounting for 31.5% of all global deaths (2). Dysregulation of the immune system triggers an early inflammatory cascade, followed by immunosuppression and long-term immune disturbances lasting for weeks and months (3, 70). Hence, the development of strategies to prevent and treat organ dysfunction to improve the prognosis of septic patients. In patients admitted to the intensive care unit (ICU), achieving 80% of the prescribed protein intake was associated with lower mortality rates (4). While the optimal timing for administering parenteral nutrition to patients with sepsis remains uncertain, it is essential to highlight the importance of optimized nutritional therapies for supporting immune function, particularly PUFAs, which are integral components of cell and organelle membrane phospholipids (5). The saturated fatty acids (SFAs) and MUFAs can be synthesized endogenously through *de novo* lipogenesis and desaturation pathways in human body. Specifically, MUFAs are generated by the desaturation of SFAs, a reaction mediated by stearoyl-CoA desaturase 1 (SCD1), which introduces a double bond at the Δ9 position to yield oleic acid (18:1 ω9) and palmitoleic acid (16:1 ω7). However, humans lack Δ12 and Δ15 fatty acid desaturases, which are essential for introducing double bonds at the n-6 and n-3 positions of fatty acid chains. Consequently, polyunsaturated fatty acids (PUFAs) such as linoleic acid (LA, 18:2 ω6) and α-linolenic acid (ALA, 18:3 ω3) cannot be synthesized endogenously and must be obtained through dietary intake, thus classified as essential fatty acids (6) (Figure 1). An increasing amount of research has shown that supplementation with PUFAs may improve the recovery of patients with sepsis, but there is yet to be a consistent conclusion (7–9). Nonetheless, observational studies are unable to fully eliminate confounding factors or reverse causation. Although several randomized controlled trials (RCTs) have reported the beneficial effects of ω-3 PUFAs in reducing mortality in sepsis (7, 10, 11), supporting evidence from other RCTs remains inconsistent (12, 13), and the sample sizes of these RCTs are relatively small. A recent meta-analysis has demonstrated that ω-3 PUFAs can reduce mortality in patients with sepsis (9). However, the efficacy of different routes of administration and doses of PUFA supplements in sepsis management appears to vary (14, 15). Therefore, the causal link between PUFAs and the sepsis risk remains inadequately established.

To address these limitations, Mendelian randomization (MR) has emerged as a powerful tool for inferring causal relationships in epidemiological research. MR leverages genetic variants as instrumental variables to mimic randomized controlled trials, reducing bias from confounding factors and reverse causation (16–18). MR offers a novel means for elucidating the role of PUFAs in sepsis. Recent studies have utilized MR to assess the influence of PUFAs on various diseases, and found that PUFAs are directly related to the risk of specific diseases, such as cardiovascular disease (19, 20), diabetes (21), chronic kidney disease (22), cancer (23), and COVID-19 (24). Observational studies have revealed that levels of palmitoleic (PA), GLA, and eicosapentaenoic acid (EPA) are decreased in patients with COVID-19, whereas those of oleic acid (OA), LA, and arachidonic acids (AA) are increased (25). In particular, the omega-3 index [measured by EPA and docosahexaenoic acid (DHA)] was significantly reduced and inversely correlated with the need for mechanical ventilation and mortality in COVID-19 patients (26, 27). This phenomenon may be attributed to the decreased production of pro-repair mediators and the subsequent exacerbation of inflammatory processes. Analysis based on the UK Biobank dataset revealed that nearly all PUFA indicators, including total PUFAs, ω-6 PUFAs, ω-3 PUFAs, LA, and DHA, except the ω-6/ω-3 ratio were inversely correlated with the future risk of severe pneumonia and COVID-19 (28). MR analysis has demonstrated that higher ω-3 PUFAs, especially DHA, may reduce COVID-19 susceptibility and severity, while also support the causal relationship between DPA/AA and lower severe COVID-19 risk (24). Consequently, MR may serve as a valuable and effective tool for evaluating the causal role of circulating PUFAs in sepsis susceptibility and severity.

A key innovation of this study is the integration of two-sample MR with animal-model validation. MR provides evidence of causal links between circulating PUFAs and sepsis susceptibility in human cohorts, while controlled experiments in animals test relevance and probe mechanisms. This combination mitigates the limitations inherent to each approach: population-level causality from MR and mechanistic and interventional insight from preclinical animal models. Identifying *PCSK9* rs11591147 as a mediator links genetic epidemiology to molecular biology and suggests a therapeutic target for sepsis prevention and treatment. The work translates population genetics into preclinical evidence to inform personalized strategies based on PUFA status and *PCSK9* genotype.

## 2 Materials and methods

### 2.1 Data sources

We conducted an observational cohort study using UK Biobank data and a two-sample MR analysis leveraging Genome-Wide Association Study (GWAS) summary statistics of UFAs and sepsis to evaluate associations between plasma UFAs and sepsis status. The UK Biobank is a large-scale prospective cohort initiative designed to elucidate the genetic and environmental determinants of human disease, with over 500,000 individuals of European ancestry recruited. In this project, a targeted high-throughput nuclear magnetic resonance (NMR) metabolomics platform (Biomarker Quantification Version 2020), developed and validated by Nightingale Health Ltd., was employed to quantify circulating plasma concentrations of ω-3 and ω-6 PUFAs in 114,999 participants. All data were obtained directly from published GWAS on human metabolic traits.^1^ Using single nucleotide polymorphisms (SNPs) related to human serum UFA levels as instrumental variables, we studied 486,484 European male and female individuals, including 12,243,539 mutation sites (GWAS ID: ieu-b-4980). Patients related to the susceptibility and severity of sepsis were obtained from the UK Biobank, including sepsis patients (case: 11,643, control: 474,841; ieu-b-4980), sepsis patients treated in critical care (case: 1,380, control: 429,985; ieu-b-4981), sepsis patients who died within 28 days of critical care (case: 347, control: 431,018; ieu-b-4982), sepsis patients who died within 28 days (case: 1,896, control: 484,588; ieu-b-5086), and sepsis patients under 75 years of age (case:11,568, control: 451,301; ieu-b-5088). In all datasets, patients and controls were included, with adjustments made for age, sex, genotyping chip, and the first 10 principal components (Supplementary Table 1). Recruitment for the UK Biobank began in 2006 and concluded in 2010, with ongoing follow-up of participants. The data for this study were extracted in 2021. The UK Biobank received ethical approval from the Research Ethics Committee (REC reference: 11/NW/0382). Informed consent was obtained from all participants. All analyses followed the STROBE-MR guidelines (16, 29).

### 2.2 Genetic variants selection criteria

Mendelian randomization (MR) analysis requires satisfaction of three core assumptions for valid causal inference s: (a) strong association between genetic instrumental variants (IVs) and circulating UFAs level; (b) independence from confounders affecting both UFAs level and sepsis; (c) the genetic IVs influence sepsis only through the UFAs level and not via other biological pathways (30). To satisfy MR assumptions, we identified SNPs significantly associated with serum UFA levels with a *p*-value less than 5 × 10^−8^. We performed linkage disequilibrium (LD) clumping the clumping process (*R*^2^ < 0.01, clumping distance = 10,000 kb) to ensure instrument independence by removing SNPs in high LD. Finally, SNPs with minor allele frequency (MAF) <0.01 were excluded, and palindromic SNPs were not included in the instrumental variables. In Mendelian randomization, the *F*-statistic is used to evaluate the strength of an instrument variable. When the *F* value exceeds 10, it is a solid instrumental variable (31).

### 2.3 Mendelian randomization analysis

We employed inverse-variance weighted (IVW) as primary analysis, supplemented by MR-Egger, weighted median, simple mode, and weighted mode—to estimate causal effects of genetically predicted serum UFA levels on sepsis susceptibility and severity. The IVW method combines Wald ratio estimates for each SNP and causal estimates using the weighted average of the Wald ratio estimates, providing the most accurate results when all selected SNPs are valid IVs. At the same time, we also performed FDR correction on the results. We used various methods for sensitivity analysis to ensure the robustness of the pleiotropic IV. Statistical significance was set *p*-value <0.05.

Horizontal pleiotropy was evaluated using MR-Egger regression and PRESSO. Instrument heterogeneity was assessed through Cochran’s *Q* statistic in both IVW and MR-Egger frameworks.

A *p*-value <0.05 represents significant heterogeneity in the IVs. Leave-one-out analysis was performed using IVW to assess the influence of individual variants on observed associations. Through Steiger analysis, reverse causal inference was conducted to explore whether susceptibility and severity of sepsis had a causal effect on UFA levels.

### 2.4 Reagents

Biochemical testing reagents, including alanine aminotransferase (ALT, LOT: 140123002), aspartate aminotransferase (AST, LOT: 140224008), triglyceride (TG, LOT: 141722012), total cholesterol (TC, LOT: 141622017), creatinine (CREA, LOT: 141124016), urea (LOT: 141322015), creatine kinase (CK, LOT: 142524011), creatine kinase-MB (CK-MB, LOT: 142623008), lactate dehydrogenase (LDH, LOT: 142724001), low density lipoprotein-cholesterol (LDL-C, LOT: 142022016), high density lipoprotein-cholesterol (HDL-C, LOT: 142122017) and glucose (LOT: 141523002), were purchased from Mindray (Shenzhen, China). Omegaven (LOT: 16SI8748) was purchased from Fresenius Kabi (Germany). Evolocumab (Cas: 1256937-27-5) was provided by TargetMol Chemicals Inc. (Boston, Massachusetts, United States).

### 2.5 Mice and interventions

The animal experiments were approved by the Ethics Committee of Qilu Hospital (approval number: DWLL-2023-176). This study strictly followed the ARRIVE guidelines (Animals in Research: Reporting *In Vivo* Experiments) humane animal treatment and complied with relevant legislation. Male *C57BL/6* mice (8–12 weeks old, Charles River Laboratories) were utilized for all experiments. The mice were housed under controlled environmental conditions (22–24 °C, 60–65% humidity, and a 12-h light/dark cycle) with ad libitum access to food and water throughout the study period. Mice were randomly divided into five groups: sham, CLP, CLP + ω-3 PUFAs, CLP + evolocumab, and CLP + ω-3 PUFAs + evolocumab. In the sham group, the abdominal cavity was opened and then sutured. Mice in the CLP group were opened in the abdominal cavity, and their cecum was ligated and punctured. Three days prior to the establishment of CLP, a solution of ω-3 PUFAs (0.5 g/kg, Omegaven^®^ LOT, 16SI8748) was administered intraperitoneally once daily until 1 h before the surgery. Additionally, evolocumab (10 mg/kg, Cas, 1256937-27-5) was injected intraperitoneally into mice 3 days before surgery and 1 h prior to surgery to create different treatment groups (5, 32).

A septic mouse model was established using the CLP method, as previously described (33). Following ≥7 days of aseptic acclimation, mice were anesthetized via inhalation of 2% isoflurane (RWD Life Science, Shenzhen, China), the abdomen of the mice was routinely disinfected using iodophor. Subsequently, a 1.5-cm midline laparotomy was performed to access the peritoneal cavity. Next, the cecum was exteriorized and carefully dissected from the mesentery using toothless forceps. Half of the free end of the cecum was ligated with 4-0 silk suture and punctured twice using a 21-gauge needle. The peritoneum, muscle tissue, and skin were closed successively using sutures. The entire experiment was conducted in the temperature range of 22–25 °C. Postoperatively, fluid resuscitation was administered via subcutaneous injection of warm sterile saline (1 mL/25 g body weight) to support hemodynamic recovery. Immediately after the operation, the mice were returned to their cages and fed separately to resume their normal diet and water intake. The 7-day survival rates of the 15 mice in each group were observed. Twenty-four hours after CLP, the mice were sacrificed under anesthesia of i.p. injection of 2% sodium pentobarbital (0.2 mL/10 g body weight; Bio-Techne, China), and their blood, hearts, livers, spleens, lungs, and kidneys were harvested.

### 2.6 Histological analysis

Twenty-four hours after the CLP or sham operation, the hearts, livers, spleens, lungs, and kidneys from mice were carefully excised and immersed in a 4% formaldehyde solution for 48 h. Following paraffin embedding, tissues were sectioned at 4 μm thickness and stained with hematoxylin and eosin (H&E) for histological assessment.

### 2.7 Biochemical testing

After centrifugation at 15,000 rpm for 15 min, the collected blood was separated into upper layer plasma for biochemical index detection. The multiple organ injury parameters ALT, AST, TG, TC, CREA, UREA, CK, CK-MB, LDH, LDL-C, HDL-C and glucose were quantified using biochemical estimation kits on an automatic analyzer (Mindray BS-240VET, Shenzhen, China) per manufacturer protocols.

### 2.8 Statistical analyses

Statistical analyses were performed using R programming (version 4.2.2). “Two Sample MR” (34) package (version 0.5.6) and the “MRPRESSO” (35) package (version 0.5.6) were used to conduct the MR analysis. Animal experimental data in this study were analyzed using GraphPad Prism software (version 9.5.1). PASS 15 was used to perform prospective sample size calculations (two-sided, *α* = 0.05, power = 0.80). The effect sizes were estimated from preliminary data obtained from two key pilot experiments conducted at the outset of the study, including the 7-days survival rate and biochemical indicator. Intergroup differences were analyzed by one-way ANOVA with Tukey’s *post-hoc* test. All data represent mean ± standard deviation (SD). The threshold for statistical significance was set at *p* < 0.05.

## 3 Results

### 3.1 Instrumental variables selection

Seventy-one SNPs associated with UFAs at *p* < 5 × 10^−8^ were selected as instrumental variables. The instrument strength was all greater than 10, indicating no evidence of weak instrumental variable bias in our analysis.

### 3.2 Two-sample MR analysis

**Figure 2:**
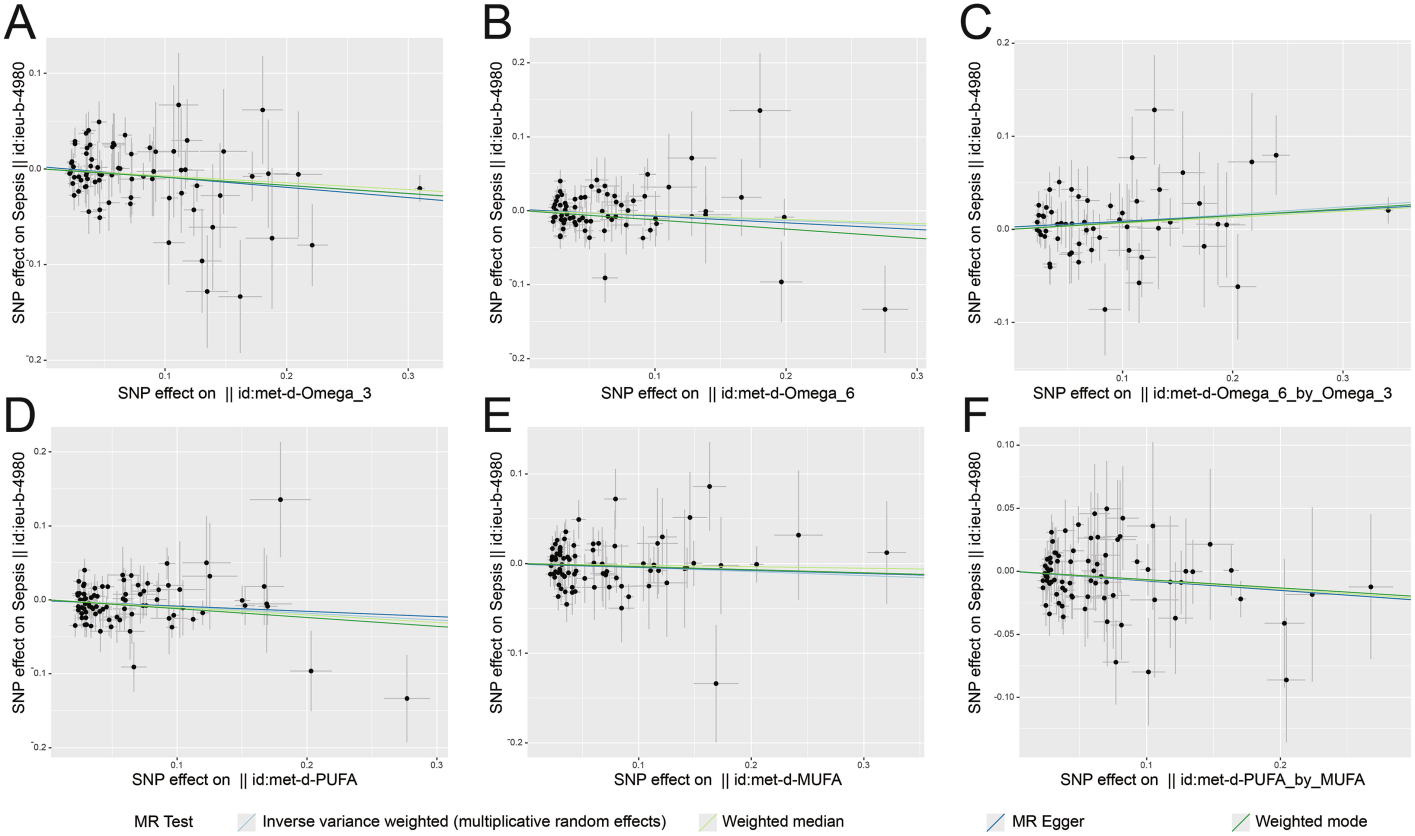
Scatter plots from genetically predicted accelerometer assessed UFAs levels of sepsis susceptibility. Genetically predicted accelerometer assessed different UFAs levels of sepsis susceptibility, respectively (**A**: ω-3 PUFAs; **B**: ω-6 PUFAs; **C**: ω-6/ω-3 ratio; **D**: PUFAs; **E**: MUFAs; **F**: PUFAs/MUFAs ratio) in sepsis patients (ieu-b-4980).

**Figure 3:**
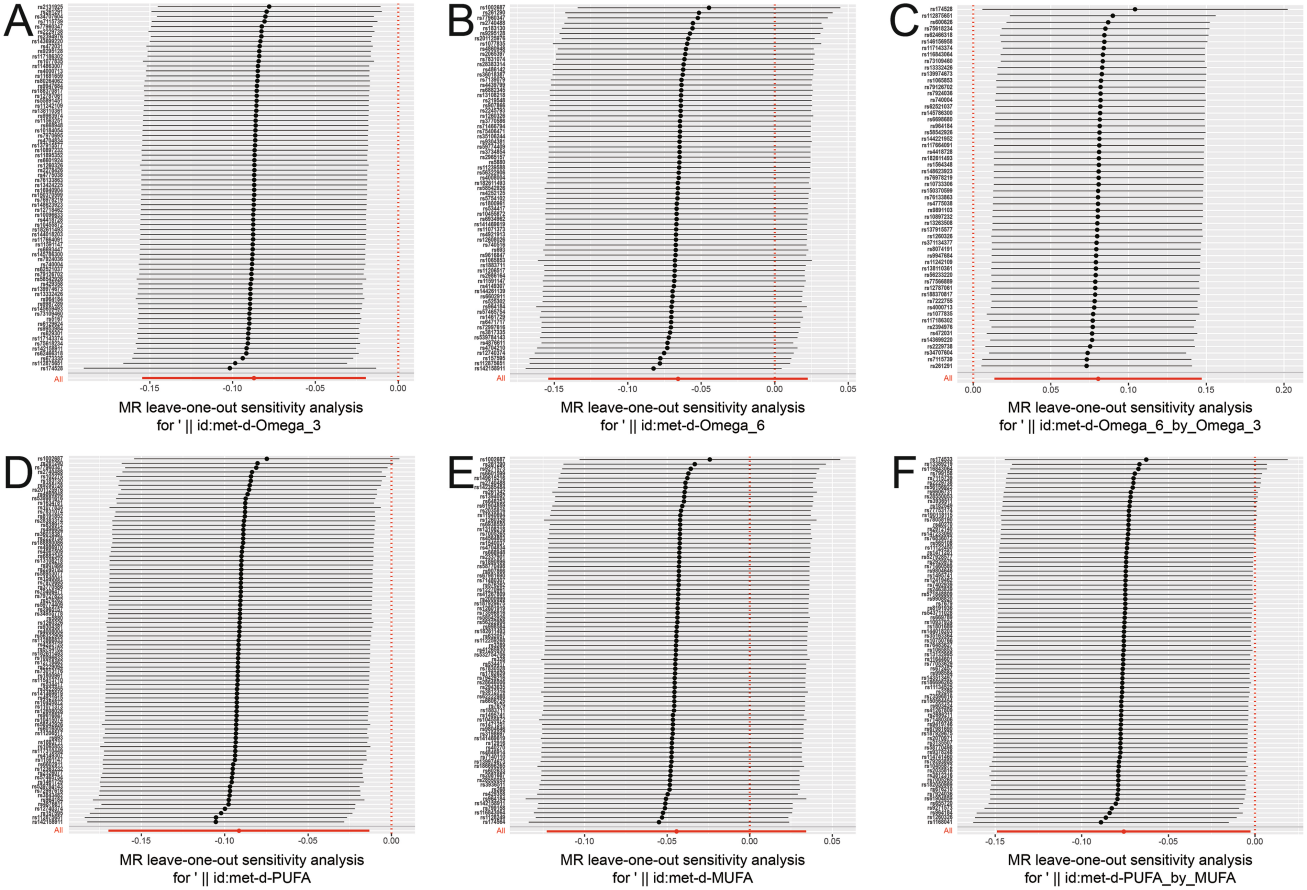
Leave one out analysis for the causal association between UFAs levels and sepsis susceptibility. Leave one out analysis assessed the causal association between different UFAs levels and sepsis susceptibility, respectively (**A**, ω-3 PUFAs; **B**, ω-6 PUFAs; **C**, ω-6/ω-3 ratio; **D**, PUFAs; **E**, MUFAs; **F**, PUFAs/MUFAs ratio) in sepsis patients (ieu-b-4980).

We analyzed the role of UFAs in sepsis using a two-sample MR approach. We observed that increasing ω-3 PUFAs (OR: 0.912, 95% CI: 0.855–0.973, *p* = 0.005), ω-6 PUFAs (OR: 0.914, 95% CI: 0.840–0.994, *p* = 0.036), total PUFAs (OR: 0.894, 95% CI: 0.830–0.963, *p* = 0.003) and PUFAs/MUFAs ratio (OR: 0.927, 95% CI: 0.862–0.997, *p* = 0.042) were associated with a low susceptibility of sepsis. The ω-6/ω-3 ratio had the opposite effect of increasing susceptibility to sepsis (OR: 1.084, 95% CI: 1.014–1.159, *p* = 0.018) and 28 days-death rates in sepsis (OR: 1.157, 95% CI: 1.001–1.337, *p* = 0.048). Meanwhile, the FDR-corrected results showed that the results of ω-3 PUFAs and total PUFAs were still significant (Table 1 and Figure 2). MR-Egger intercept and MR-PRESSO showed no evidence of horizontal pleiotropy (Supplementary Table 2). Cochran’s *Q* statistic of MR-Egger and IVW revealed no significant heterogeneity among the UFA-associated genetic IVs for sepsis risk in the sepsis GWAS (Supplementary Table 3). The leave-one-out analysis indicated that the results were reliable (Figure 3). Collectively, these findings demonstrated robust data consistency with minimal evidence of objective bias.

**Table 1:** Association of UFAs genetic IVs with sepsis susceptibility and severity.

GWAS ID	Outcome ID	nsnp	*b*	SE	*p*-value	FDR	OR	95% CI
met-d-Omega_3	ieu-b-4980	71	−0.092	0.033	0.005^*^	0.030^#^	0.912	(0.855–0.973)
	ieu-b-4981	72	−0.220	0.168	0.189	0.381	0.802	(0.578–1.114)
	ieu-b-4982	73	−0.132	0.084	0.115	0.144	0.876	(0.744–1.033)
	ieu-b-5086	73	−0.134	0.074	0.068	0.144	0.874	(0.757–1.010)
	ieu-b-5088	74	−0.058	0.031	0.057	0.143	0.943	(0.888–1.002)
met-d-Omega_6	ieu-b-4980	69	−0.090	0.043	0.036^*^	0.180	0.914	(0.840–0.994)
	ieu-b-4981	71	−0.267	0.217	0.219	0.365	0.765	(0.500–1.172)
	ieu-b-4982	71	−0.025	0.111	0.823	0.827	0.975	(0.784–1.213)
	ieu-b-5086	71	−0.022	0.100	0.827	0.827	0.978	(0.804–1.191)
	ieu-b-5088	71	−0.059	0.040	0.140	0.350	0.942	(0.871–1.020)
met-d-Omega_6_by_Omega_3	ieu-b-4980	56	0.080	0.034	0.018^*^	0.090	1.084	(1.014–1.159)
	ieu-b-4981	56	0.195	0.174	0.261	0.261	1.215	(0.865–1.708)
	ieu-b-4982	56	0.147	0.087	0.089	0.148	1.159	(0.978–1.373)
	ieu-b-5086	56	0.146	0.074	0.048^*^	0.120	1.157	(1.001–1.337)
	ieu-b-5088	56	0.042	0.031	0.171	0.214	1.043	(0.982–1.108)
met-d-PUFA	ieu-b-4980	81	−0.112	0.038	0.003^*^	0.015^#^	0.894	(0.830–0.963)
	ieu-b-4981	83	−0.341	0.198	0.085	0.142	0.711	(0.482–1.048)
	ieu-b-4982	83	−0.114	0.099	0.248	0.302	0.892	(0.735–1.083)
	ieu-b-5086	83	−0.095	0.092	0.302	0.302	0.909	(0.759–1.089)
	ieu-b-5088	83	−0.070	0.037	0.058	0.142	0.932	(0.867–1.002)
met-d-MUFA	ieu-b-4980	80	−0.042	0.040	0.294	0.460	0.959	(0.887–1.037)
	ieu-b-4981	80	−0.201	0.223	0.368	0.460	0.818	(0.529–1.266)
	ieu-b-4982	80	−0.072	0.107	0.503	0.503	0.931	(0.754–1.149)
	ieu-b-5086	76	−0.105	0.092	0.256	0.460	0.900	(0.751–1.079)
	ieu-b-5088	80	−0.050	0.036	0.172	0.460	0.951	(0.886–1.022)
met-d-PUFAs_by_MUFA	ieu-b-4980	79	−0.076	0.037	0.042^*^	0.210	0.927	(0.862–0.997)
	ieu-b-4981	79	−0.023	0.214	0.915	0.915	0.977	(0.642–1.488)
	ieu-b-4982	79	−0.041	0.107	0.700	0.875	0.960	(0.778–1.184)
	ieu-b-5086	79	−0.096	0.096	0.318	0.530	0.908	(0.752–1.097)
	ieu-b-5088	79	−0.042	0.037	0.257	0.530	0.959	(0.893–1.031)

UFAs, unsaturated fatty acids; IVs, instrumental variants; GWAS, genome-wide association study; PUFAs, polyunsaturated fatty acids; MUFAs, monounsaturated fatty acids; nsnp, number of single nucleotide polymorphisms; SE, standard error; FDR, false discovery rate; OR, odds ratio; 95% CI, 95% confidence interval. ieu-b-4980: sepsis; ieu-b-4981: sepsis with 28-day death in critical care; ieu-b-4982: sepsis with critical care; ieu-b-5086: sepsis with 28-day death; ieu-b-5088: sepsis under 75 years old; ^*^p-value <0.05 represents significant difference, ^#^FDR <0.05 represents significant difference.

### 3.3 SNPs in sepsis patients related to PUFAs

**Figure 4:**
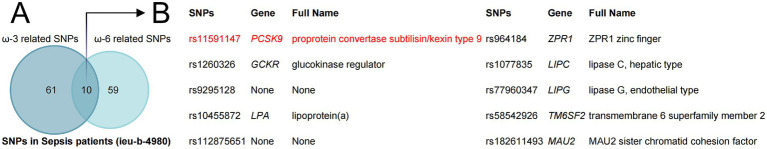
Related SNPs in sepsis patients (ieu-b-4980). **(A)** Seven-one SNPs were related to ω-3 PUFAs, 69 SNPs were associated with ω-6 PUFAs. Ten SNPs were both related to ω-3 and ω-6 PUFAs. **(B)** Ten SNPs loci and related gene names.

Even high levels of ω-3 and ω-6 PUFAs are protective against sepsis, and a higher ω-6/ω-3 ratio has the opposite effect. To explore the role of SNPs in the relationship between ω-3 and ω-6 PUFAs in patients with sepsis (ieu-b-4980), we conducted further SNPs analysis. Ten SNPs related to the two-sample MR analysis were associated with both ω-3 and ω-6 PUFAs. Six SNPs were only related to ω-3 PUFAs and 59 SNPs were only associated with ω-6 PUFAs (Figure 4). Among the 10 SNPs listed in Supplementary Table 4, proprotein convertase subtilisin/kexin type 9 (*PCSK9*), glucokinase regulator (*GCKR*), lipoprotein(a) (*LPA*), ZPR1 zinc finger (*ZPR1*), lipase C, hepatic type (*LIPC*), lipase G, endothelial type (*LIPG*), transmembrane 6 superfamily member 2 (*TM6SF2*), and MAU2 sister chromatid cohesion factor (*MAU2*) have been reported to be associated with lipid metabolism disorders. Therefore, heightened clinical vigilance for sepsis susceptibility is warranted in this high-risk population.

### 3.4 Supplementing ω-3 PUFAs and inhibiting PCSK9 can alleviate sepsis-induced damage

**Figure 5:**
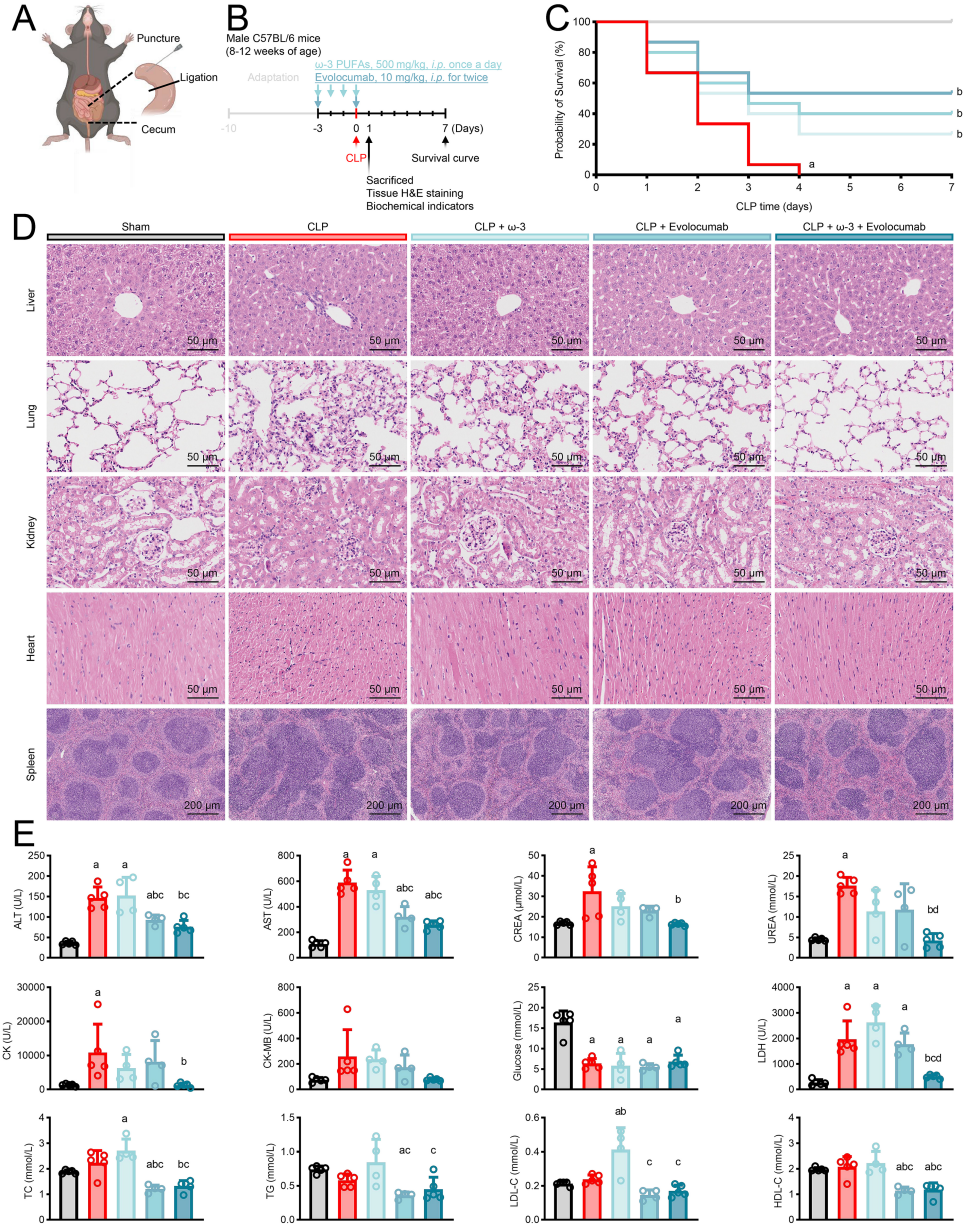
Supplementing ω-3 PUFAs and inhibiting PCSK9 can alleviate sepsis-induced damage. **(A)** Establishment of CLP model for sepsis in mice. **(B)** ω-3 PUFAs and evolocumab administration methods. **(C)** Seven-day survival curve of septic mice (*N* = 15 per group). **(D)** Mice were exposed to CLP surgery for 24 h, and then organs were excised and subjected to H&E staining. Representative H&E images of liver, heart, spleen, lung, and kidney in sham, CLP, CLP + ω-3 PUFAs, CLP + evolocumab, CLP + ω-3 PUFAs + evolocumab groups (*N* ≥ 4 per group). **(E)** Twenty-four hours after CLP surgery, blood of mice was collected and centrifuged to obtain plasma for measuring ALT, AST, TG, TC, CREA, urea, CK, CK-MB, LDH, LDL-C, HDL-C and glucose (*N* ≥ 4 per group). Data are shown as the mean ± SD from ≥4 independent experiments. a, vs. sham *p* < 0.05; b, vs. CLP *p* < 0.05; c, vs. CLP + ω3 *p* < 0.05; d, vs. CLP + evolocumab. *p <* 0.05 represents significant difference (one-way ANOVA test). Partial illustrations were generated using BioRender.com. A valid publication license has been acquired.

To further demonstrate the outcomes of two-sample MR analysis, a gold standard sepsis mouse CLP model was employed. Prior to establishing the sepsis model, we administered or did not administer ω-3 PUFAs or evolocumab to mice to mimic the levels of PUFAs and *PCSK9* gene mutation in the pre-disease state of patients (Figures 5A,B).

Exogenous supplementation of ω-3 PUFAs or inhibition of PCSK9, or a combination, contributed to a reduction in the 7-day mortality rate among septic mice (73.3, 60.0, and 46.7%, respectively). Moreover, when these two treatments were administered simultaneously, the mortality rate tended to decrease further (Figure 5C). Through tissue H&E staining and the detection of biochemical indicators related to multiple organ injuries, we discovered that supplementation with ω-3 PUFAs and inhibition of PCSK9 more effectively alleviated damage to various tissues, reduce infiltration of inflammatory cells, and prevented the loss of organ function caused by sepsis (Figures 5D,E). It is possible that the protective effect of ω-3 PUFAs in sepsis is mediated by mutations in *PCSK9*.

## 4 Discussion

In this study, we clarified the causal relationship between UFAs and sepsis susceptibility using MR analysis. High circulating PUFAs, especially ω-3 PUFAs, may reduce the susceptibility to sepsis. Animal experiments have also demonstrated that the combination of ω-3 PUFAs and PCSK9 monoclonal antibodies can reduce mortality and multiorgan damage in CLP septic mice.

In recent years, although enhanced understanding of sepsis pathogenesis, improved clinical phenotyping and advances in organ support modalities, the pathogenesis of sepsis still needs to be clarified, and treatment methods are limited. PUFAs have received considerable attention owing to their powerful immunomodulatory effects, such as enhancing the immune response to infections and promoting inflammation regression to avoid tissue damage. Emerging evidence indicates that PUFAs demonstrate therapeutic potential in sepsis. Supplementation of PUFAs with enteral nutrition or parenteral nutrition may improve the healing of patients with sepsis, but there is yet to be a completely consistent conclusion. Pontes-Arruda et al. (7) found that sepsis patients fed a diet enriched with EPA experienced a significant reduction in 28 days-mortality. Diet-enriched EPA can also significantly improve the oxygenation status, resulting in more ventilator-free days, more ICU-free days, and fewer new organ dysfunctions. However, an RCT by Rice et al. (8) showed that enteral supplementation of ω-3 PUFAs did not improve ventilator-free days and 60-day hospital mortality in patients with acute lung injury and may be harmful. A meta-analysis (9) of 17 RCTs (*n* = 1,239) showed ω-3 PUFAs nutritional supplement may reduce ICU length of stay and mechanical ventilation, but not mortality in sepsis patients, which is not enough to justify the routine use of ω-3 PUFAs in the management of sepsis. Recently, Giamarellos-Bourboulis (36) summarized previous clinical findings and indicated that ω-3 PUFAs exert protective effects in sepsis, ranging from prevention to early treatment. ω-3PUFAs attenuate systemic inflammation by inhibiting the production of pro-inflammatory eicosanoids and promoting the synthesis of specialized pro-resolving mediators. Fayyaz et al. (37) demonstrated that ω-3 PUFAs can regulate microRNA expression, thereby modulating lipid metabolism. Advances in personalized nutrition have provided a promising avenue for more effective disease treatment strategies. Our findings are consistent with the above viewpoints and further extend these observations by identifying a causal relationship between circulating ω-3 PUFAs and reduced sepsis susceptibility, and by further demonstrating that this protective effect is enhanced by PCSK9 inhibition. Although the levels of PUFAs in patients with sepsis is unclear, our MR analysis indicated that high circulating ω-3 PUFAs were associated with reduced susceptibility to sepsis, and a high ω-6/ω-3 ratio was associated with increased susceptibility to sepsis and 28 days-death rates in sepsis. A delicate balance between ω-3 mediated anti-inflammation and ω-6 PUFA-mediated pro-inflammation is essential to balance the inflammatory response during physiological processes. Biologically active ω-6 AA activates inflammatory reactions, and uncontrolled inflammatory reactions lead to immune damage (38). In contrast, ω-3 PUFAs (EPA/DHA) competitively inhibit the oxidation of LOX to AA and reduce the release of AA from the cell membrane, thereby reducing the production of metabolites from AA. Meanwhile, metabolites of EPA/DHA also competitively bind to the same receptor of PGE2/TXA2/LTB4, but their ability to cause inflammation is significantly reduced, which helps to inhibit inflammatory reactions and regulate immunity. AA can be released from the membrane into the cell cytoplasm by phospholipase A2 (39, 40). In terms of physiology, most released AA rapidly recombine into membrane phospholipids, making fatty acids unsuitable for use as oxidation substrates (41). Thus, under “rest” conditions, basal eicosanoid production maintains homeostasis through regulated physiological signaling. However, in the presence of stress (e.g., inflammatory irritants), sufficient amounts of AA are released to drive a significant increase in eicosanoids, such as PGD2, PGE2, and four series of LTs, increases and acts as mediators and regulators of inflammatory reactions (42).

Current evidence establishes that specific AA-derived metabolites, notably lipoxin A4 (LXA4), serve as potent specialized pro-resolving mediators that actively terminate inflammatory responses (43, 44). Intriguingly, the eicosanoids produced in the early stages of inflammation are related to later induction of resolution. These considerations complicated our understanding of the role of ω-6 PUFAs in general and AA, particularly in the context of inflammation (45). Although our results suggest that ω-6 PUFAs may reduce the occurrence of sepsis (OR, 0.914; 95% CI: 0.840–0.994; *p* = 0.036), it is important to acknowledge that the marginal significance of this finding may lead to overinterpretation. Furthermore, a higher ratio of ω-3 PUFAs to total PUFAs appears to play a more critical role in the context of sepsis. The inconsistency of the studies may be due to the patients being in different stages of sepsis, different amounts of nutritional supplements, different autoimmune statuses, and individual genetic differences. ω-3 PUFAs nutritional supplements may reduce immune resistance to microorganisms in the inflammatory initiation stage of sepsis, and ω-6 PUFAs may improve the immune killing ability by promoting inflammation. Therefore, PUFAs may play different roles in different stages of sepsis. Acceptable individualized treatment should be reflected in the proportion of ω-6/ω-3 PUFAs.

In our study, 10 SNPS with common correlations were identified by merging SNPS in different traits. Several SNPS, such as rs11591147 in *PCSK9*, rs1260326 in *GCKR*, rs10455872 in *LPA*, rs964184 in *ZPR1*, rs58542926 in *TM6SF2*, and rs182611493 in *MAU2* have been clinically associated with multiple metabolic disorders, including coronary heart disease, hyperlipidemia, non-alcoholic fatty liver disease (46–51). rs1077835of *LIPC* and rs77960347 of *LIPG*, have also recently been reported to be associated with high plasma lipid levels (52, 53).

In sepsis, PCSK9 degrades low-density lipoprotein (LDL) receptors in hepatocytes and very low-density lipoprotein (VLDL) receptors in adipocytes, thereby diminishing pathogenic lipid uptake and clearance. Concurrently, it may promotes cholesterol accumulation and potentiate Toll-like receptor hyperresponsiveness in macrophages, amplifying inflammatory responses (54). Furthermore, PCSK9 inhibitors, novel lipid-lowering medications, have shown promising effects in various diseases and can significantly reduce the risk of major adverse cardiovascular events (55). Nevertheless, it remains uncertain whether the benefits of PCSK9 inhibition are associated with circulating PUFAs levels in patients with sepsis (56, 57). The rs11591147 mutation in *PCSK9*, a loss-of-function variant, was causally linked to reduced LDL cholesterol and attenuated coronary heart disease risk (51, 58, 59). In sepsis, high plasma PCSK9 levels are associated with acute organ failure and increased mortality (32, 60–62). Surprisingly, the presence of mutant alleles of *PCSK9*, including the rs11591147 mutation, reduced the risk of death from sepsis (63), although the underlying mechanism of this phenomenon remains to be elucidated. In our study, the causal role of the reduced susceptibility to sepsis by ω-3 fatty acids was connected to the SNP of *PCSK9*. Therefore, we hypothesized that *PCSK9* loss-of-function would enhance the protective effect of ω-3 PUFAs supplementation against sepsis. In this study, we conducted animal experiments to validate the findings of two-sample MR analysis. We found that inhibiting PCSK9 in conjunction with administering exogenous ω-3 PUFAs, further reduced the 7-day mortality rate in septic mice. Although the results did not reach statistical significance, this trend was evident (Figure 5C). This combination therapy also reduced liver, kidney, and other tissue damage caused by sepsis (Figures 5D,E). Therefore, when supplementing ω-3 PUFAs for patients with sepsis, the *PCSK9* gene background should also be considered, as it may confer greater benefits to patients with specific *PCSK9* gene mutations.

As a key protein regulating intracellular glucose disposal, GCKR is expressed in most hepatocytes and participates in glucose metabolism and lipogenesis (64, 65). SNP rs1260326 of *GCKR* is well established to be associated with non-alcoholic fatty liver disease and type 2 diabetes (49, 50, 66), but not in sepsis. In contrast to ω-6 PUFAs, a significant interaction was observed between rs1260326 and high ω-3 PUFAs levels in serum triglycerides concentrations (67). However, it is worth exploring whether the rs1260326 mutation can cause an increase in triglyceride levels in sepsis patients with high ω-3 PUFAs levels. Therefore, it is necessary to understand the role of *GCKR* polymorphisms in sepsis-associated lipid metabolism disorders.

These findings carry potential clinical relevance. Our MR analysis implicated circulating ω-3 PUFAs as a protective factor against sepsis, implying that dietary ω-3 supplementation could reduce sepsis risk in high risk populations. Moreover, the *PCSK9* rs11591147 variant emerges as a mediator, offering a novel genetic biomarker of sepsis susceptibility. Early genotyping and PUFA assessment may enable risk stratification and targeted prevention in vulnerable individuals. Collectively, these results support a precision medicine approach to sepsis that integrates genetic background with metabolic profile. Importantly, conclusions derived from genetic analyses and animal studies; translational validation in humans is needed. This study has some limitations. First, as GWAS come from European ancestry, it may not be possible to promote it to other populations. The metabolism of UFAs may vary among different populations, genetic backgrounds, environments, and dietary habits, which requires careful consideration of the applicability of the results of this study in different populations. Second, owing to the use of public data, the lack of data prevented us from conducting ω-3 or ω-6 PUFAs metabolite interactions with sepsis. Third, our findings integrate genetic associations and preclinical evidence, but require validation in large prospective cohorts of sepsis with available PUFA biomarkers.

This study has some limitations, which also point to future research directions. First, GWAS data derive from European ancestry; evaluating diverse populations is necessary to assess generalizability. Second, future studies should investigate the role of specific PUFA metabolites (e.g., resolvins and protectins from EPA/DHA) in sepsis, as these mediators may be key contributors to the anti-inflammatory activity. Third, large prospective cohorts with available PUFA biomarkers and *PCSK9* genotype data are needed to validate our findings in clinical settings. Fourth, mechanistic studies should explore the molecular pathways through which PCSK9 modulates the protective effect of ω-3 PUFAs, such as lipid metabolism, inflammatory signaling, or immune cell function. Fifth, trials should evaluate combined ω-3 PUFA supplementation and PCSK9 inhibition, especially in *PCSK9* variant carriers. Such work will clarify translational potential and guide trial design.

## 5 Conclusion

Mendelian randomization analysis and experimental validation indicated that in patients with *PCSK9* gene mutations, high levels of circulating polyunsaturated fatty acids (PUFAs), particularly ω-3 PUFAs, may reduce susceptibility to sepsis. Conversely, a higher proportion of ω-6 PUFAs among total PUFAs may increase the risk of sepsis. Therefore, early detection of PUFA levels and *PCSK9* genotypes, along with the application of ω-3 PUFA nutritional supplements in susceptible populations, may help reduce the risk of developing sepsis.

## Data Availability

Publicly available datasets were analyzed in this study. This data can be found here: all data relevant to this study are included in the article and Supplementary material. Additionally, summary data for sepsis can be accessed from the UK Biobank. The summary data for the UFAs are available at https://gwas.mrcieu.ac.uk/.

## Footnotes

1
https://gwas.mrcieu.ac.uk/
