# Supplementary material for: Protective impact of circulating ω-3 PUFAs on sepsis susceptibility through PCSK9 variant: evidence from Mendelian randomization and animal experiments
Source: Front Med (Lausanne). 2026 Jan 13;12:1701207. doi: 10.3389/fmed.2025.1701207 (PMC12834780; doi:10.3389/fmed.2025.1701207)
Supplement: Supplementary file 1 [file Table_1.DOCX]

**Supplementary information**

**CONTENTS**

[**Supplemental table 1. Data sources and characteristics of used studies in our study.** 2](#_Toc198542946)

[**Supplemental table 2. Horizontal pleiotropy test of UFAs genetic IVs in sepsis GWAS.** 4](#_Toc198542947)

[**Supplemental table 3. Heterogeneity test of UFAs genetic IVs in sepsis GWAS.** 5](#_Toc198542948)

[**Supplemental table 4. SNPs in Sepsis patients (ieu-b-4980) related to ω-3 PUFAs and ω-6 PUFAs.** 7](#_Toc198542949)

**Supplemental table 1. Data sources and characteristics of used studies in our study.**

| **GWAS ID** | **Year** | **Population** | **Sex** | **Number of case** | **Number of control** | **Number of SNPs** | **Adjustments** | [**Trait**](https://gwas.mrcieu.ac.uk/datasets/?gwas_id__icontains=ieu-b-4981&year__iexact=&trait__icontains=&consortium__icontains=&sort=-trait) |
| --- | --- | --- | --- | --- | --- | --- | --- | --- |
| ieu-b-4980 | 2021 | European | Males and Females | 11,643 | 474,841 | 12,243,539 | Adjusted for age, sex, chip, and the first 10 PCAs | Sepsis：The sepsis diagnosis adhered to the criteria outlined in the International Classification of Diseases (ICD)-9 and ICD-10 codes as delineated in the previously published Global Burden of Disease (GBD) study. |
| ieu-b-4981 | 2021 | European | Males and Females | 347 | 431,018 | 12,243,324 | Adjusted for age, sex, chip, and the first 10 PCAs | Sepsis (28 day death in critical care): The diagnosis of sepsis meets the standards of ICD-9 and ICD-10, and the patient died within 28 days after admission to critical care. |
| ieu-b-4982 | 2021 | European | Males and Females | 1,380 | 429,985 | 12,243,372 | Adjusted for age, sex, chip, and the first 10 PCAs | Sepsis (critical care): The diagnosis of sepsis meets the standards of ICD-9 and ICD-10, and the patient admitted to critical care. |
| ieu-b-5086 | 2021 | European | Males and Females | 1,896 | 484,588 | 12,243,487 | Adjusted for age, sex, chip, and the first 10 PCAs | Sepsis (28 day death): The diagnosis of sepsis meets the standards of ICD-9 and ICD-10, and the patient died within 28 days. |
| ieu-b-5088 | 2021 | European | Males and Females | 11,568 | 451,301 | 12,243,540 | Adjusted for age, sex, chip, and the first 10 PCAs | Sepsis (under 75): The sepsis diagnosis adhered to the criteria outlined in the International Classification of Diseases (ICD)-9 and ICD-10 codes as delineated in the previously published Global Burden of Disease (GBD) study. And the patients who had a code for sepsis before the age of 75. |
| met-d-Omega_3 | 2020 | European | Males and Females | 114,999 | | 12,321,875 | NA | Omega-3 fatty acids |
| met-d-Omega_6 | 2020 | European | Males and Females | 114,999 | | 12,321,875 | NA | Omega-6 fatty acids |
| met-d-Omega_6_by_Omega_3 | 2020 | European | Males and Females | 114,999 | | 12,321,875 | NA | Ratio of omega-6 fatty acids to omega-3 fatty acids |
| met-d-PUFA | 2020 | European | Males and Females | 114,999 | | 12,321,875 | NA | Polyunsaturated fatty acids |
| met-d-MUFA | 2020 | European | Males and Females | 114,999 | | 12,321,875 | NA | Monounsaturated fatty acids |
| met-d-PUFA_by_MUFA | 2020 | European | Males and Females | 114,999 | | 12,321,875 | NA | Ratio of polyunsaturated fatty acids to monounsaturated fatty acids |
| **Note:** GWAS: genome-wide association study; SNPs: number of single‐nucleotide polymorphism, NA: not available | | | | | | | | |

**Supplemental table 2. Horizontal pleiotropy test of** **UFAs genetic IVs in sepsis GWAS.**

| **GWAS ID** | **Outcome ID** | **MR Egger-intercept** | **SE** | ***P*-value** | **MR-PRESSO** |
| --- | --- | --- | --- | --- | --- |
| **met-d-Omega_3** | ieu-b-4980 | 0.000 | 0.004 | 0.995 | 0.251 |
|  | ieu-b-4981 | 0.014 | 0.020 | 0.474 | 0.595 |
|  | ieu-b-4982 | 0.018 | 0.010 | 0.071 | 0.943 |
|  | ieu-b-5086 | 0.014 | 0.008 | 0.111 | 0.324 |
|  | ieu-b-5088 | -0.003 | 0.004 | 0.397 | 0.177 |
| **met-d-Omega_6** | ieu-b-4980 | 0.001 | 0.004 | 0.871 | 0.090 |
|  | ieu-b-4981 | 0.030 | 0.023 | 0.189 | 0.553 |
|  | ieu-b-4982 | 0.016 | 0.012 | 0.186 | 0.364 |
|  | ieu-b-5086 | 0.005 | 0.011 | 0.632 | 0.160 |
|  | ieu-b-5088 | 0.003 | 0.004 | 0.490 | 0.150 |
| **met-d-PUFA** | ieu-b-4980 | -0.002 | 0.004 | 0.568 | 0.129 |
|  | ieu-b-4981 | 0.011 | 0.021 | 0.608 | 0.568 |
|  | ieu-b-4982 | 0.005 | 0.010 | 0.631 | 0.489 |
|  | ieu-b-5086 | 0.003 | 0.010 | 0.754 | 0.112 |
|  | ieu-b-5088 | 0.000 | 0.004 | 0.963 | 0.112 |
| **met-d-MUFA** | ieu-b-4980 | 0.000 | 0.004 | 0.965 | 0.080 |
|  | ieu-b-4981 | 0.007 | 0.021 | 0.745 | 0.156 |
|  | ieu-b-4982 | 0.011 | 0.010 | 0.296 | 0.290 |
|  | ieu-b-5086 | -0.009 | 0.009 | 0.308 | 0.336 |
|  | ieu-b-5088 | 0.001 | 0.003 | 0.744 | 0.314 |
| Note: IVs, instrumental variants; UFAs, unsaturated fatty acids; GWAS, genome-wide association study; PUFAs, polyunsaturated fatty acids; MUFAs, monounsaturated fatty acids; MR Egger-intercept, Mendelian Randomization Egger’s intercept; SE, standard error; MR-PRESSO, Mendelian Randomization Pleiotropy Residual Sum and Outlier test. P-value > 0.05 represents no significant pleiotropy. | | | | | |

**Supplemental table 3. Heterogeneity test of UFAs genetic IVs in sepsis GWAS.**

| **GWAS ID** | **Outcome ID** | **Method** | **Cochran's Q statistic** | **Q_df** | **Q_*P*-value** |
| --- | --- | --- | --- | --- | --- |
| **met-d-Omega_3** | ieu-b-4980 | MR Egger | 88.944 | 69 | 0.053 |
|  |  | IVW | 88.944 | 70 | 0.063 |
|  | ieu-b-4981 | MR Egger | 68.119 | 70 | 0.541 |
|  |  | IVW | 68.638 | 71 | 0.557 |
|  | ieu-b-4982 | MR Egger | 51.308 | 71 | 0.962 |
|  |  | IVW | 54.664 | 72 | 0.936 |
|  | ieu-b-5086 | MR Egger | 74.620 | 71 | 0.361 |
|  |  | IVW | 77.356 | 72 | 0.312 |
|  | ieu-b-5088 | MR Egger | 83.474 | 72 | 0.167 |
|  |  | IVW | 84.317 | 73 | 0.172 |
| **met-d-Omega_6** | ieu-b-4980 | MR Egger | 84.344 | 67 | 0.075 |
|  |  | IVW | 84.377 | 68 | 0.087 |
|  | ieu-b-4981 | MR Egger | 66.693 | 69 | 0.556 |
|  |  | IVW | 68.454 | 70 | 0.530 |
|  | ieu-b-4982 | MR Egger | 71.691 | 69 | 0.389 |
|  |  | IVW | 73.546 | 70 | 0.363 |
|  | ieu-b-5086 | MR Egger | 82.640 | 69 | 0.125 |
|  |  | IVW | 82.917 | 70 | 0.139 |
|  | ieu-b-5088 | MR Egger | 81.585 | 69 | 0.143 |
|  |  | IVW | 82.155 | 70 | 0.152 |
| **met-d-Omega_6_by_**  **Omega_3** | ieu-b-4980 | MR Egger | 69.334 | 54 | 0.078 |
|  |  | IVW | 69.743 | 55 | 0.087 |
|  | ieu-b-4981 | MR Egger | 54.250 | 54 | 0.465 |
|  |  | IVW | 54.811 | 55 | 0.482 |
|  | ieu-b-4982 | MR Egger | 46.250 | 54 | 0.764 |
|  |  | IVW | 47.192 | 55 | 0.764 |
|  | ieu-b-5086 | MR Egger | 46.894 | 54 | 0.742 |
|  |  | IVW | 47.808 | 55 | 0.743 |
|  | ieu-b-5088 | MR Egger | 56.805 | 54 | 0.371 |
|  |  | IVW | 59.155 | 55 | 0.326 |
| **met-d-PUFA** | ieu-b-4980 | MR Egger | 94.435 | 79 | 0.113 |
|  |  | IVW | 94.828 | 80 | 0.123 |
|  | ieu-b-4981 | MR Egger | 79.440 | 81 | 0.528 |
|  |  | IVW | 79.706 | 82 | 0.551 |
|  | ieu-b-4982 | MR Egger | 81.146 | 81 | 0.474 |
|  |  | IVW | 81.379 | 82 | 0.499 |
|  | ieu-b-5086 | MR Egger | 98.353 | 81 | 0.092 |
|  |  | IVW | 98.473 | 82 | 0.104 |
|  | ieu-b-5088 | MR Egger | 98.656 | 81 | 0.089 |
|  |  | IVW | 98.658 | 82 | 0.101 |
| **met-d-MUFA** | ieu-b-4980 | MR Egger | 97.579 | 78 | 0.066 |
|  |  | IVW | 97.582 | 79 | 0.077 |
|  | ieu-b-4981 | MR Egger | 91.636 | 78 | 0.139 |
|  |  | IVW | 91.761 | 79 | 0.154 |
|  | ieu-b-4982 | MR Egger | 84.207 | 78 | 0.295 |
|  |  | IVW | 85.400 | 79 | 0.292 |
|  | ieu-b-5086 | MR Egger | 78.862 | 74 | 0.328 |
|  |  | IVW | 79.986 | 75 | 0.325 |
|  | ieu-b-5088 | MR Egger | 84.513 | 78 | 0.288 |
|  |  | IVW | 84.630 | 79 | 0.312 |
| **met-d-PUFAs_by_**  **MUFA** | ieu-b-4980 | MR Egger | 70.649 | 77 | 0.682 |
|  |  | IVW | 70.649 | 78 | 0.710 |
|  | ieu-b-4981 | MR Egger | 74.490 | 77 | 0.560 |
|  |  | IVW | 75.955 | 78 | 0.544 |
|  | ieu-b-4982 | MR Egger | 62.225 | 77 | 0.889 |
|  |  | IVW | 62.305 | 78 | 0.903 |
|  | ieu-b-5086 | MR Egger | 86.009 | 77 | 0.226 |
|  |  | IVW | 87.266 | 78 | 0.221 |
|  | ieu-b-5088 | MR Egger | 77.521 | 77 | 0.462 |
|  |  | IVW | 78.335 | 78 | 0.468 |
| **Note:** UFAs, unsaturated fatty acids; IVs, instrumental variants; GWAS, genome-wide association study; PUFAs, polyunsaturated fatty acids; MUFAs, monounsaturated fatty acids; IVW, inverse variance weighted; MR, Mendelian randomization; Q_df, degrees of freedom of Q statistic, Q_*P*-value, *P*-value of Q statistic. Q_*P*-value > 0.05 represents no significant heterogeneity. | | | | | |

**Supplemental table 4. SNPs in Sepsis patients (ieu-b-4980) related to ω-3 PUFAs and ω-6 PUFAs.**

|  | **SNPs** | **Gene** | **Full Name** | **Location** | **effect_**  **allele** | **other_**  **allele** |
| --- | --- | --- | --- | --- | --- | --- |
| 1 | rs11591147 | *PCSK9* | proprotein convertase subtilisin/kexin type 9 | Chromosome 1 | T | G |
| 2 | rs1260326 | *GCKR* | glucokinase regulator | Chromosome 2 | C | T |
| 3 | rs9295128 | None | None | Chromosome 6 | T | G |
| 4 | rs10455872 | *LPA* | lipoprotein(a) | Chromosome 6 | G | A |
| 5 | rs112875651 | None | None | Chromosome 8 | A | G |
| 6 | rs964184 | *ZPR1* | ZPR1 zinc finger | Chromosome 11 | C | G |
| 7 | rs1077835 | *LIPC* | lipase C, hepatic type | Chromosome 15 | G | A |
| 8 | rs77960347 | *LIPG* | lipase G, endothelial type | Chromosome 18 | G | A |
| 9 | rs58542926 | *TM6SF2* | transmembrane 6 superfamily member 2 | Chromosome 19 | T | C |
| 10 | rs182611493 | *MAU2* | MAU2 sister chromatid cohesion factor | Chromosome 19 | G | A |
| **Note:** SNPs: number of single‐nucleotide polymorphism. | | | | | | |
